# Diphtheria—Its Causes, Prevention and Proper Treatment

**Published:** 1879-03

**Authors:** 


Diphtheria—Its Causes, Prevention and Proper Treatment. By J. H. Kellogg, M.D., Member of the Am. Pub. Health Ass'n; the Am. Society of Microscopy; the Michigan State Med. Ass'n; author of numerous works on health, etc., etc. Published by the Good Health Publishing Co., Battle Creek, Mich.

Diphtheria is a disease of widespread fatality, and any information that may be presented by careful observers should be read by both practitioners and patients. This monograph treats of the history, pathology and treatment of the disease, and should be read by all desirous of obtaining detailed information on the subject of which it treats.

